# Effects of Aging and Hypercholesterolemia on Oxidative Stress and DNA Damage in Bone Marrow Mononuclear Cells in Apolipoprotein E-deficient Mice

**DOI:** 10.3390/ijms14023325

**Published:** 2013-02-05

**Authors:** Clarissa L. Tonini, Bianca P. Campagnaro, Lis P. S. Louro, Thiago M. C. Pereira, Elisardo C. Vasquez, Silvana S. Meyrelles

**Affiliations:** 1 Laboratory of Transgenes, Health Sciences Center, Federal University of Espirito Santo, Vitoria, ES 29043-900, Brazil; E-Mails: clarissatonini@gmail.com (C.L.T.); biancacampagnaro@yahoo.com.br (B.P.C.); lislouro@terra.com.br (L.P.S.L.); evasquez@pq.cnpq.br (E.C.V.); 2 Federal Institute of Education, Science and Technology (IFES), Vila Velha, ES 29106-010, Brazil; E-Mail: pereiratmc@gmail.com; 3 Department of Pharmaceutical Sciences, University of Vila Velha (UVV), Vila Velha, ES 29102-770, Brazil; 4 Emescam School of Health Sciences, Vitoria, ES 29045-402, Brazil

**Keywords:** DNA damage, oxidative stress, comet assay, genotoxicity, apolipoprotein E

## Abstract

Recent evidence from apolipoprotein E-deficient (apoE^−/−^) mice shows that aging and atherosclerosis are closely associated with increased oxidative stress and DNA damage in some cells and tissues. However, bone marrow cells, which are physiologically involved in tissue repair have not yet been investigated. In the present study, we evaluated the influence of aging and hypercholesterolemia on oxidative stress, DNA damage and apoptosis in bone marrow cells from young and aged apoE^−/−^ mice compared with age-matched wild-type C57BL/6 (C57) mice, using the comet assay and flow cytometry. The production of both superoxide and hydrogen peroxide in bone marrow cells was higher in young apoE^−/−^ mice than in age-matched C57 mice, and reactive oxygen species were increased in aged C57 and apoE^−/−^ mice. Similar results were observed when we analyzed the DNA damage and apoptosis. Our data showed that both aging and hypercholesterolemia induce the increased production of oxidative stress and consequently DNA damage and apoptosis in bone marrow cells. This study is the first to demonstrate a functionality decrease of the bone marrow, which is a fundamental extra-arterial source of the cells involved in vascular injury repair.

## 1. Introduction

Atherosclerosis is a progressive disease that results from lipid disorders, enhanced oxidative stress and inflammation [1,2]. There is cumulative evidence that elevated levels of either molecular oxygen or chemical derivatives of oxygen, *i.e.*, reactive oxygen species (ROS), can damage different types of molecules, [3] and tissues, including cardiovascular tissues [4]. For example, excessive aging-associated ROS production leads to lipid oxidation, vascular cell damage, atherogenesis and vascular remodeling [5–8]. DNA is another cellular target of ROS, resulting in detrimental effects, including the activation of inflammation, cell apoptosis and aging in diverse tissues and organs [9,10].

The apolipoprotein E-deficient (apoE^−/−^) mouse, which spontaneously develops hypercholesterolemia and atherosclerotic lesions [2,11], has contributed to research concerning the role of oxidative stress in atherosclerosis. The phenotypes of the apoE^−/−^ mouse include age-dependent vascular senescence [12] increased vascular production of ROS [13,14], endothelial dysfunction [15] and apoptosis [14].

Recent studies in apoE^−/−^ mice have shown that DNA oxidation increases in an age-dependent manner in the liver and whole blood cells but not in the plaque-rich aorta segments or lung tissues [16,17], indicating a tissue-specific phenomenon. In parallel, oxidative stress and DNA damage in bone marrow mononuclear cells (MNC), which are physiologically involved in tissue repair [18] and have therapeutic vascular effects in hypercholesterolemia and atherosclerosis [13,14], have not yet been investigated in the apoE^−/−^ mouse model.

The comet assay, which has been used to analyze age-related DNA damage in several cell types in apoE^−/−^ mice [16,17], is a suitable technique for the quantitative measurement of DNA genotoxicity in any tissue. Similarly, flow cytometry is an appropriate technique for the detection of ROS production [19] to evaluate the effects of aging and hypercholesterolemia on oxidative stress, DNA damage and apoptosis. Despite these advances, the effects of these events in bone marrow MNC have not yet been investigated in the murine model of spontaneous hypercholesterolemia and atherosclerosis.

Therefore, the aim of this study was to examine the hypothesis that aging and hypercholesterolemia are associated with the enhanced production of ROS, DNA damage and apoptosis in bone marrow MNC from wild-type C57 and apoE^−/−^ mice.

## 2. Results and Discussion

**Figure 1:**
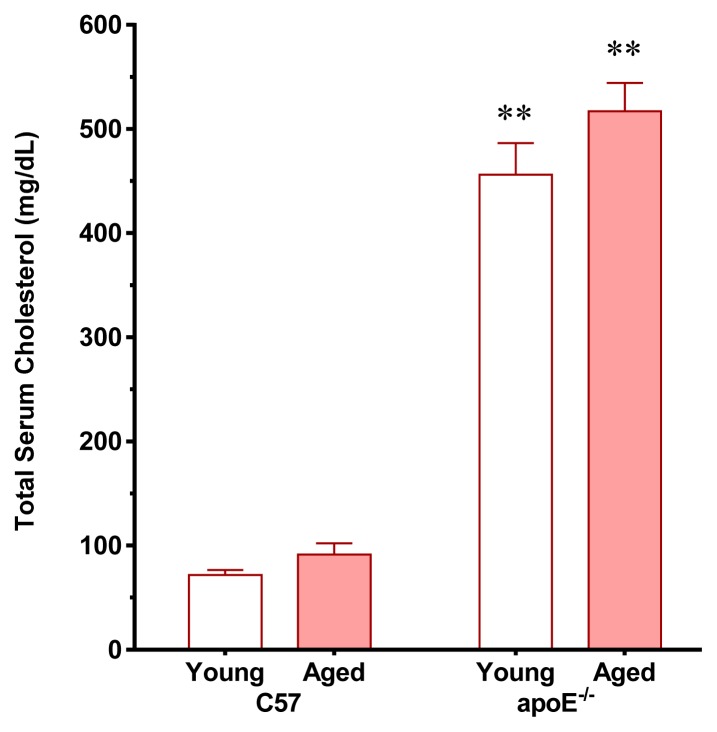
Total serum cholesterol levels in young and aged apoE^−/−^ mice compared with age-matched C57 mice fed a regular chow diet. The values are presented as means ± SEM. ** *p*< 0.01 *vs.* the age-matched C57 group.

The daily chow and water intake was similar between the two age-matched strains of mice and there was a significant age-dependent weight gain in C57 and apoE^−/−^ mice. As expected, consistent with classical and recent studies [17,20,21], the total serum cholesterol levels were significantly higher in young apoE^−/−^ mice (6.4-fold) than in age-matched C57 mice (71 ± 12 mg/dL, *p* < 0.01) without significant age-related differences in the total serum cholesterol levels in the two strains of mice (Figure 1). Aiming to exclude metabolic parameters that could be affected by aging and particularly by atherosclerosis, we also measured blood glucose and observed that the values were similar in young and aged C57 (75 ± 2 *vs.* 89 ± 6 mg/dL, respectively) and apoE^−/−^ (95 ± 12 *vs.* 108 ± 9 mg/dL, respectively) mice. There is evidence that under a normal diet aging does not affect other biochemical profiles in the apoE^−/−^ mouse [22,23].

Several studies [2,12,13,15,24] have shown that chronic injuries to the arterial wall in apoE^−/−^ mice contribute to the development of atherosclerosis. Moreover, a complex repair system that involves both local and bone marrow-derived cells is required to maintain arterial homeostasis and integrity [13,14,24]. Thus, the results obtained in the present study might clarify how hypercholesterolemia and aging might compromise the viability of the bone marrow to produce the vascular progenitor cells responsible for arterial repair. However, when the normal function is perturbed, arterial wall cells are no longer replaced [24], which can be quantified by the senescence technique [12].

### 2.1. Vascular Senescence and Lipid Deposition

**Figure 2:**
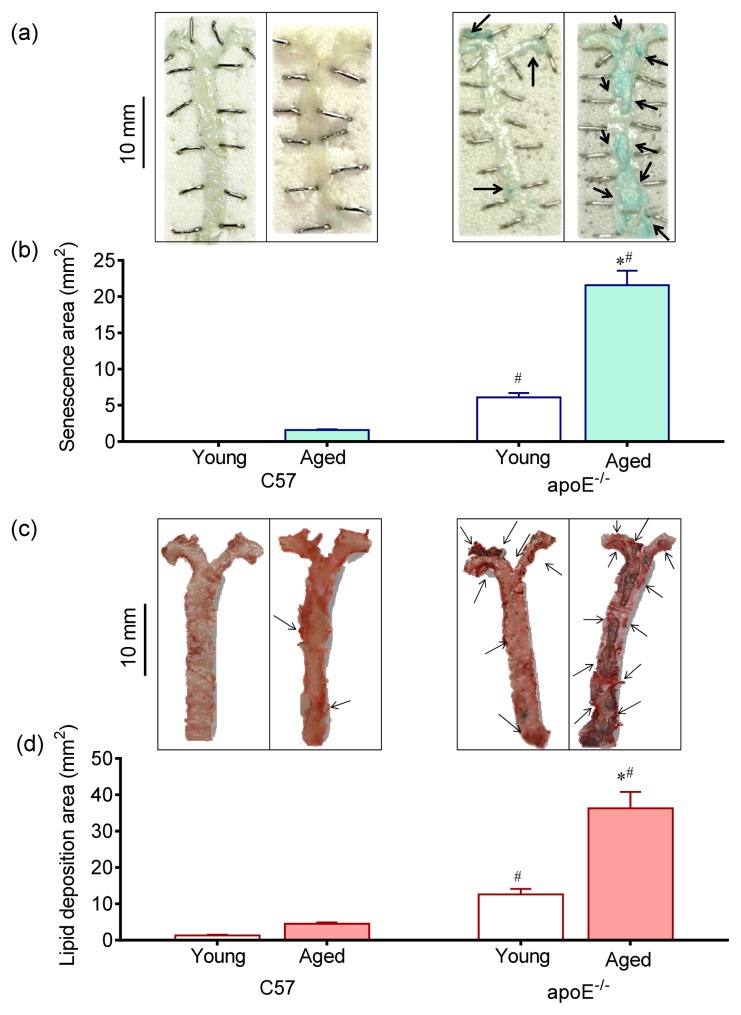
Vascular lipid deposition and senescence. Representative aorta *en face* macrographs of (**a**) senescence (X-gal, pH 6.0) and (**c**) lipid deposition (Oil-Red-O staining) in young and aged C57 mice compared with age-matched apoE^−/−^ mice. A remarkable large area of vascular senescence and lipid deposition was observed in aged apoE^−/−^ mice. Bar graphs show average vascular senescence (**b**) and lipid deposition (**d**) areas. The values are presented as means ± SEM. * *p* < 0.05 *vs.* the respective young group, ^#^*p* < 0.05 *vs.* the age-matched C57 group.

The Figure 2 shows *en face* analysis of vascular senescence through β-gal activity, which is considered to be one of the best markers currently available. As observed in representative images in Figure 2a and summarized in Figure 2b bar graph, a remarkable increase in stained area was observed in the aorta of aged apoE^−/−^ compared with young apoE^−/−^ mice (21.6 ± 2.1 and 6.1 ± 0.6 mm^2^, respectively *p* < 0.05) but not in age-matched C57 mice (1.6 ± 0.080 and 0.005 ± 0.001 mm^2^, respectively). Senescent cells show progressive DNA damage, telomere shortening, negative gene expression regulators and increased proinflammatory molecules contributing to both progression of atherosclerosis and pathogenesis of vascular aging [2,15].

As illustrated in Figure 2c and summarized in Figure 2d bar graph, *en face* whole aorta analysis showed a greater lipid deposition area in aged apoE^−/−^ mice compared with young apoE^−/−^ mice (36.3 ± 4.5 and 12.6 ± 1.5 mm^2^, *p* < 0.05) and compared with age-matched C57 mice (4.5 ± 0.4 and 1.3 ± 0.2 mm^2^, *p* < 0.05). The above results are in agreement with previous study from our laboratory showing the association of aging with both senescence and lipid deposition in the apoE^−/−^ mouse [12].

### 2.2. Reactive Oxygen Species (ROS)

**Figure 3:**
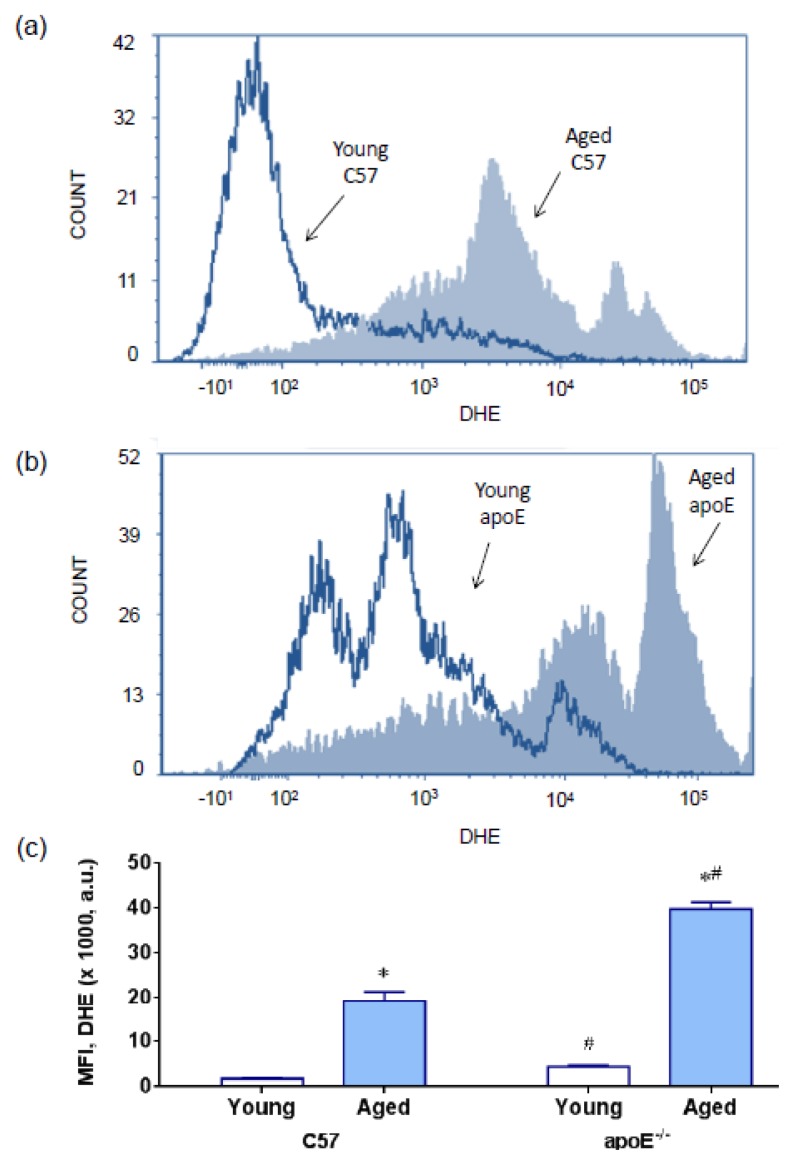
Production of superoxide anions. Representative histograms from flow cytometric analysis using dihydroethidium (DHE) in young and aged C57 mice (**a**) compared with age-matched apoE^−/−^ mice (**b**); the log fluorescence (X axis) illustrates the intensity of fluorescence for the number of cells counted (note the higher scales in the apoE histogram). A remarkable increase in the level of superoxide anions was observed in aged mice mainly in apoE^−/−^ mice. (**c**) Bar graph showing mean fluorescence intensity (MFI). The values are presented as means ± SEM. * *p* < 0.05 *vs.* the respective young group, ^#^*p* < 0.05 *vs.* the age-matched C57 group.

**Figure 4:**
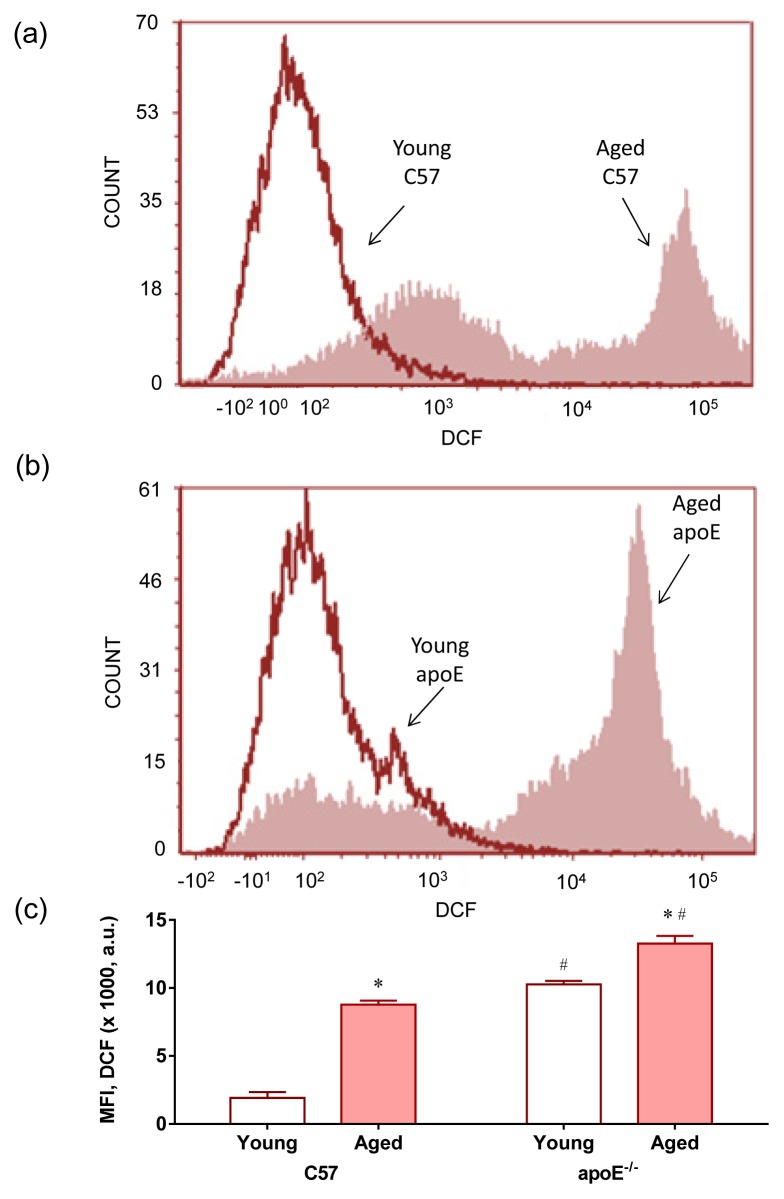
Production of hydrogen peroxide. Representative histogram from flow cytometric analysis with 2′,7′-dichlorofluorescein (DCF) in young and aged C57 mice (**a**) compared with age-matched apoE^−/−^ mice (**b**); the log fluorescence (X-axis) illustrates the intensity of fluorescence for the number of cells counted (note the higher scales in the apoE^−/−^ histogram). Increased levels of hydrogen peroxide were observed in aged mice mainly in apoE^−/−^ mice; (**c**) Bar graph showing mean fluorescence intensity (MFI). The values are presented as the means ± SEM. * *p* < 0.05 *vs.* the respective young group, ^#^*p* < 0.05 *vs.* the age-matched C57 group.

The term oxidative stress is often used to imply a condition in which cells are exposed to excessive levels of either molecular oxygen or chemical derivatives of oxygen (termed as ROS) [25]. We evaluated the production of ROS using flow cytometry with dihydroethidium (DHE) to quantify the production of superoxide anions (Figure 3) and 2′,7′-dichlorofluorescein (DCF) to quantify the production of hydrogen peroxide (Figure 4); the presence of both compounds was indicated by the median fluorescence intensity (in a.u.). Typical histograms from flow cytometric analysis show a rightward-shift in the log of DHE fluorescence in aged C57 (Figure 3a) and apoE^−/−^ mice (Figure 3b) compared with their respective young counterparts; a rightward-shift was also shown in young apoE^−/−^ mice compared with age-matched wild type C57 mice. As summarized in Figure 3c, we observed a remarkable increase in the levels of superoxide anions in aged hypercholesterolemic animals (C57: 19 ± 3 *vs.* apoE^−/−^: 40 ± 2 × 10^3^ a.u., *p* < 0.05) compared with young mice (C57: 2.3 ± 0.3 *vs.* apoE^−/−^: 8.7 ± 1.5 × 10^3^ a.u., *p* < 0.05). Similarly, the production of hydrogen peroxide (Figure 4c) was significantly increased in aged hypercholesterolemic animals (C57: 8.7 ± 0.5 *vs.* apoE^−/−^: 13.2 ± 0.5 × 10^3^ a.u., *p* < 0.05) compared with young mice (C57: 1.9 ± 0.4 *vs.* apoE^−/−^: 10.4 ± 0.2 × 10^3^ a.u., *p* < 0.05). Typical histograms from flow cytometric analysis with DCF are shown in Figure 4 a,b and summarized in Figure 4c.

ROS can be produced from intracellular oxygen radicals mainly formed through cytosolic NADPH oxidases [26], by leakage from the mitochondrial respiratory chain and by some enzymes [15,27,28]. In the apoE^−/−^, it has been shown that the induction of enzyme systems including xanthine oxidase [29], uncoupled eNOS [30], lipoxygenase [31] and NADPH oxidase [32] contributes to ROS formation in all layers of the diseased arterial wall, particularly in atherosclerotic plaques, in the apoE^−/−^ mouse model [13,15]. To our knowledge, this study is the first to report a pronounced ROS production in the bone marrow cells in this murine model, including young mice. This finding supports the hypothesis that hypercholesterolemia might induce oxidative stress in nonspecific tissues.

In addition, the ROS produced during aging might exceed the antioxidant capacity of the cell [33], reflecting the increased oxidative stress observed in aged apoE^−/−^ mice. Several studies have shown marked changes in oxidative stress in multiple cell types, including neuronal and vascular cells, in aged *vs.* young apoE^−/−^ mice [15,34,35]. However, the impact of aging on ROS production in bone marrow cells had not been previously described in apoE^−/−^ mice. Our results support the idea that hypercholesterolemia and aging additively contribute to oxidative stress in bone marrow cells, leading to deleterious effects, such as DNA damage, which is a target of ROS [9,16,17].

ROS promotes DNA damage through the addition of double bonds or the removal of hydrogen atoms from DNA bases, forming a series of adducts, such as 8-oxo-G, an oxidized form of guanine that indicates oxidative DNA damage [16,36]. Moreover, ROS are potent activators of poly(ADP-ribose) polymerase (PARP), as a consequence of damage DNA. These deleterious events occur in the telomeric and nontelomeric regions of both mitochondrial and nDNA [37,38] in vessel smooth muscle cells and macrophages in plaques [36,38]. In the present study, we examined the hypothesis that DNA damage is a consequence of ROS production to determine whether these events also occur in bone marrow cells.

### 2.3. Analysis of DNA Damage (Comet Assay)

Studies have shown that cellular targets for oxidative modification through ROS include lipids, proteins and particularly DNA [39]. To combat the harmful effects of ROS, living cells have acquired a number of defenses [39]. However, when ROS production is dramatically increased under conditions such as hypercholesterolemia and aging, these repair systems do not mitigate all damages, ultimately leading to DNA fragmentation. In the present study, we analyzed this phenomenon using the alkaline comet assay, which is an easy, rapid, inexpensive and sensitive method that has potential for general applicability [40]. This assay quantifies single strand breaks and other damage that affects the migration of DNA from the nucleus under alkaline electrophoretic conditions [41]. For the comet assay analysis, an imaging system is used, which considers both the length of DNA migration and the intensity in the comet tail to calculate the score of DNA damage [42]. Comets without clearly defined heads or with most of the DNA contained within the tail were excluded from the image analysis.

**Figure 5:**
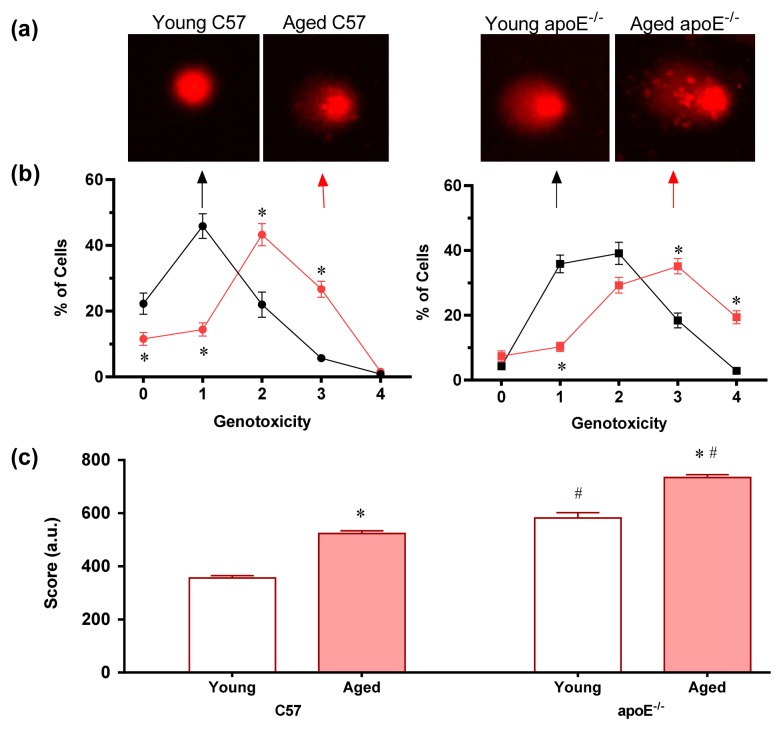
Detection of DNA damage in individual bone marrow MNC assessed by comet assay. (**a**) Typical comets showing higher DNA fragmentation in aged mice compared to young mice mainly in apoE^−/−^ mice; (**b**) Line graphs of average percentages of cells for each of the genotoxicity levels, showing higher percentage of cells at higher level of genotoxicity in aged mice, which was aggravated in hypercholesterolemic mice; (**c**) Bar graph showing the total score of MNC with DNA damage. Values are means ± SEM. * *p* < 0.05 *vs.* respective young group, ^#^*p* < 0.05 *vs.* age-matched C57 group.

We used a five-category classification system to assign values for DNA migration ranging from 0 (no damage) to 4 (almost all DNA is present in the tail). Figure 5a shows representative comets for each of the four groups of mice, showing age-related DNA damage in wild-type C57 mice and both age- and hypercholesterolemia-related DNA damage in apoE^−/−^ mice. As summarized in Figure 5b, the percentage of cells without DNA damage (level 0) was significantly higher in normocholesterolemic young wild-type C57 mice than in the other groups. Aging occurred concomitantly with higher levels of genotoxicity (peak level 2, *p* < 0.05) in C57 mice. However, in apoE^−/−^ mice, both hypercholesterolemia and aging negatively affected the level of genotoxicity, *i.e.*, hypercholesterolemic young apoE^−/−^ animals exhibited higher levels of genotoxicity than age-matched C57 mice and aging aggravated the genotoxicity levels, with a lower percentage of cells exhibiting genotoxicity levels of 0 and 1 and a higher percentage of cells exhibiting genotoxicity levels of 3 and 4, respectively (*p* < 0.05). The total DNA damage is summarized in Figure 5c. Aged C57 mice showed higher levels of DNA damage than young C57 mice (575 ± 24 *vs.* 356 ± 12 a.u., respectively). The deleterious effects of aging were observed in apoE^−/−^ mice, with animals exhibiting an aggravation in the DNA damage due to hypercholesterolemia (aged: 734 ± 12 a.u. *vs.* young: 525 ± 4 a.u., *p* < 0.05).

The comet assay confirmed the hypothesis concerning the additive effect of hypercholesterolemia and aging on oxidative stress through the observation of intense DNA breaks in aged apoE^−/−^ mice compared with the other groups. These results are the first to show that bone marrow cells present DNA damage proportional to the rate of ROS production, supporting our “cause and effect” hypothesis. These data corroborate a previous study in which Folkmann *et al.* [16] observed high levels of oxidized DNA in apoE^−/−^ mice compared with their wild-type counterparts. However, these authors also reported that the level of oxidized DNA increased in an age-dependent manner in the liver but not in the plaque-rich aorta segments or lung tissues. To reconcile the contradictory data, we propose that this is tissue-specific and does not require any situation the concurrence of aging and hypercholesterolemia. To confirm this hypothesis, recent studies in our laboratory using the same murine model showed that, at least in whole blood cells, DNA damage requires the concurrence of aging and hypercholesterolemia [17].

Despite these differences, the results obtained in the present show that hypercholesterolemia can promote DNA damage, e.g., young hypercholesterolemic apoE^−/−^ mice showed higher levels of DNA damage in bone marrow MNC than age-matched wild-type C57 mice (38% higher, *p* < 0.05). The influence of hypercholesterolemia on DNA damage has recently been observed in rats [43] and rabbits [44], including reduced oxidative DNA during dietary lipid lowering [45]. Additionally, we might also consider the relevance of ROS in this process, as recent studies show that cholesterol oxidation through ROS results in the formation of hydroperoxides, which might induce modifications and mutations in DNA [46]. Although the comet assay is consistent with DNA damage, future studies are necessary to further characterize the specific DNA damage process observed in MNC.

The accumulation of DNA damage in bone marrow cells might reflect both ongoing damage-inducing stimuli and defects in the repair machinery [16,35]. Therefore, when DNA damage is too extensive for repair or when the repairing cascades are impaired, cellular senescence and apoptosis occurs. Oxidative DNA damage induces apoptosis for genomic mechanisms such as the p53 pro-apoptotic transcriptional activity [38,45,47,48] or non-genomic resulting from mitochondrial DNA damage (more vulnerable due to lack of protective histones), compromising the energy supply and consequently apoptosis [49,50]. On the other hand, the comet assay does not allow us to detect damage in mitochondrial DNA because these molecules are small (about 17 kb) and are not attached to the nuclear matrix. As soon as lysis starts, the mtDNA begins to disperse, and by the time electrophoresis begins virtually no mtDNA molecules are left [51].

### 2.4. Apoptosis

Aside from DNA damage through ROS, it is possible that other intracellular events occur in parallel, such as the degradation of oxidative lipids (peroxidation) [46], the oxidation of amino acid side chains, protein fragmentation [52–54], the loss of intracellular calcium homeostasis and consequently, the alteration of several metabolic pathways [55–57]. Because all these processes have been documented through exhaustive apoptotic models in many different cell systems [53,56–59], we also propose that hypercholesterolemia and aging trigger the apoptosis, contributing to the senescence of bone marrow cells. Notably, although multiple cell death processes occur in atherosclerosis such as necrosis, autophagy and apoptosis, apoptosis is the only event that has been associated with senescence [38].

**Figure 6:**
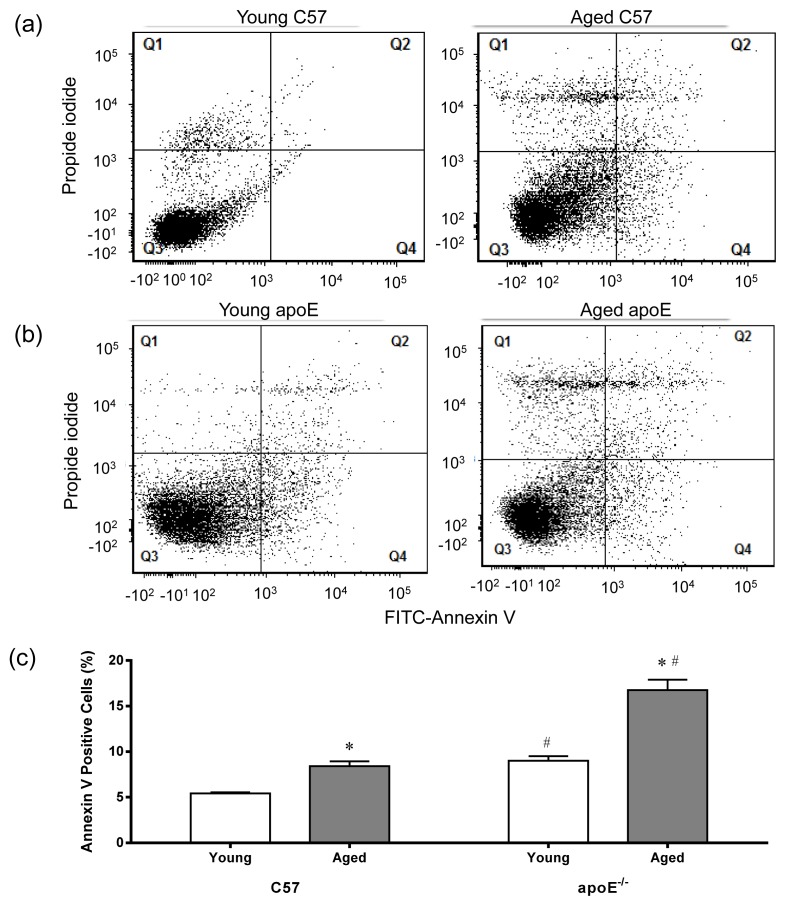
Flow cytometric analysis of apoptosis. Typical dot plots showing apoptosis ratio comparing C57 mice (**a**) with age-matched apoE^−/−^ mice (**b**) using propidium iodide (PI) and FITC-annexin V. Q1 quadrant represents damaged cells (PI positive and annexin negative). Q2 quadrant represents cell that are in late apoptosis or already dead (necrotic cells), *i.e.*, are both annexin and PI positive). Q3 quadrant represents viable cells, *i.e.*, cells that are both annexin and PI negative. Q4 quadrant represents cells in early apoptosis (cell apoptosis) annexin positive and PI negative. Note a remarkable increase in apoptotic cells number (Q2 + Q4) in the aged animals. (**c**) Bar graph shows average percentage of apoptotic cells (Q2 + Q4). Values are means ± SEM. * *p* < 0.05 *vs.* the respective young group.

We evaluated apoptosis in MNC through flow cytometry using FITC-annexin V and propidium iodide to facilitate the quantification of cell damage (Q1) and the identification of late apoptotic cells/necrotic cells (Q2), viable cells (Q3) and early apoptotic cells (Q4) [60]. The annexin V protein binds to phosphatidylserine on the surface of apoptotic cells. Figure 6 shows representative dot plots for each of the four groups of animals showing a remarkable increase in apoptotic cell number (Q2 + Q4) in aged C57 (Figure 6a) and apoE^−/−^ mice (Figure 6b). As shown in Figure 6c, both aged C57 and apoE^−/−^ mice showed an increased percentage of apoptotic cells (9.6 ± 0.6% and 21.3 ± 4.8%, respectively, *p* < 0.05) compare with young C57 and apoE^−/−^ mice (5.6 ± 0.6% and 6.0 ± 1.2%, respectively, *p* < 0.05).

Many studies have reported that the atheroprotective property of the bone marrow is “exhausted” with aging and prolonged exposure to risk factors (e.g., hypercholesterolemia) in apoE^−/−^ mice, resulting in the disequilibrium between reparative endothelial cells and inflammatory leukocytes, thereby compromising the balance of injury and repair [61–63]. The application of annexin V/propidium iodide staining demonstrated the increased apoptosis of bone marrow cells in aged animals, which was further aggravated through hypercholesterolemia (see above the results of ROS production and DNA damage). Thus, the present study supports the idea that impaired vascular repair in apoE^−/−^ mice might be secondary to the obsolescence of bone marrow cells [61], contributing to the progression of atherosclerosis. In addition, these results indicate the importance of the viability of bone marrow cells to autologous transplantation, as recently demonstrated by the efficacy of cells from healthy animals [13,14] but not consistently from diseased individuals [64,65].

## 3. Experimental Section

### 3.1. Animals

Experiments were performed in male wild-type C57BL/6 (C57, *n* = 48) and apolipoprotein E-deficient (apoE^−/−^, *n* = 24) mice bred and maintained in the animal care facility at the Laboratory of Transgenes in the Health Sciences Center at the Federal University of Espirito Santo, Brazil. The mice were housed in individual plastic cages with a controlled temperature (22–23 °C) and humidity (60%) and were exposed to a 12:12-h light-dark cycle. All mice were fed a standard chow diet and had access to water *ad libitum*. The mice were distributed into 2 groups of young (2-month-old) C57 (*n* = 12) and apoE^−/−^ (*n* = 12) mice and 2 groups of aged (18-month-old) C57 (*n* = 12) and apoE^−/−^ (*n* = 12) mice. All experimental procedures were performed in accordance with the guidelines for the care and handling of laboratory animals as recommended by the National Institutes of Health (NIH), and study protocols were previously approved by the Institutional Animal Care Committee (CEUA-Emescam, Protocol # 014/2011).

### 3.2. Analysis of Plasma Cholesterol and Glucose

Total serum cholesterol and glycemia were obtained in blood samples. Total plasma cholesterol was determined using commercial colorimetric assay kits (Bioclin, Belo Horizonte, Brazil). Glycemia was measured through a digital glucose meter (Prestige IQ, Home Diagnostics, Fort Lauderdale, FL, USA).

### 3.3. Analysis of Senescence and Vascular Lipid Deposition

Mice were euthanized with sodium thiopental overdose (100 mg/kg, IP) and perfused via the left ventricle with phosphate-buffered saline (PBS, pH 7.4; 0.1 M). Briefly, *en face* aortic samples were incubated for 24 h at 37 °C in freshly prepared β-gal staining solutions (pH 6.0) containing 2.4 mM 5-bromo-4-chrolo-3-indlyl-d-galactopyranoside (X-gal, Sigma-Aldrich, St. Louis, MO, USA), 4.7 mmol/L potassium ferrocyanide, 4.9 mmol/L potassium ferricyanide, 150 mmol/L NaCl, 1 mmol/L MgCl_2_ and 40 mmol/L citric acid. The senescence analysis was performed by detection of blue color produced by the enzymatic reaction and the images were captured with a photographic camera (Canon, USA). Then, the same samples were stained with Oil-Red-O (Sigma-Aldrich) to detect neutral lipids followed by images capture for recording. The quantification of senescence and lipid deposition was performed using a National Institutes of Health (NIH) Image program (Image-J 1.35 d, NIH, Bethesda, MD, USA) and the examiner was blinded to the experimental groups.

### 3.4. Isolation of Bone Marrow Mononuclear Bone Marrow Cells for Comet Assay and Flow Cytometry

Mice were euthanized with a sodium thiopental overdose (100 mg/kg, i.p.) and bone marrow samples were collected from femurs and tibias which were dissected and cleaned of all soft tissue. After removing epiphyses and gaining access to the marrow cavities, whole bone marrow was flushed out using a 26-gauge needle attached to a 1 mL syringe filled with Dulbecco’s Modified Eagle Medium (DMEM; Sigma, St. Louis, MO, USA). Mononuclear cells (MNC) were isolated by density-gradient centrifugation, in which the suspension of the whole bone marrow in 4 mL DMEM was loaded on 4 mL Histopaque 1083 (Sigma-Aldrich) and centrifuged for 30 min at 400 g. The MNC fraction was subsequently collected, washed in phosphate-buffered saline (PBS) and a small volume of the resulting suspension was mixed with trypan blue (0.4%) to perform a cell count and viability analysis. Lymphocytes and undifferentiated cells were analyzed using a Neubauer chamber.

**Figure 7:**
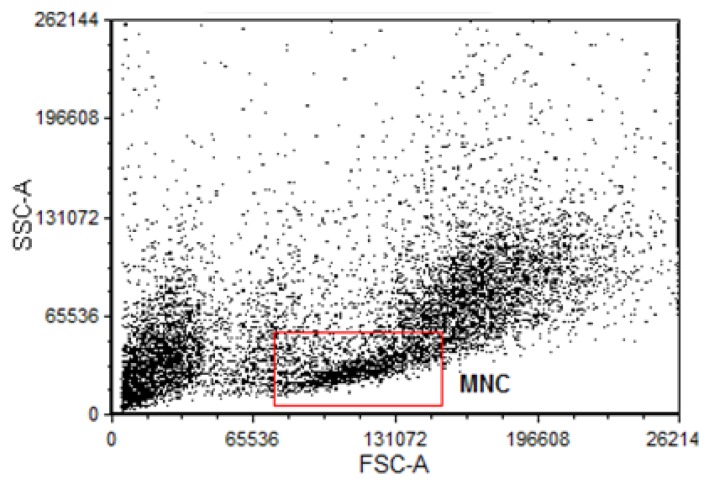
Flow cytometric histogram used to analysis of mononuclear cells (MNC). Typical dot plot representing (in linear scale) the forward scatter (FSC, correlates with the cell volume) *vs.* side scatter (SSC, correlates with granularity of the cell). The square (red) gate indicates the selected portion of data which matches with MNC and that was used in the analysis of oxidative stress (Figure 3 and Figure 4). A Total of 10,000 events were acquired for analysis.

For flow cytometry experiments, after bone marrow was flushed out with DMEM cells suspension was lysed with lysing Buffer 1X (Amersham) for 5 min at 37 °C to remove erythrocyte. After, cells suspension was centrifuged for 10 min at 1200 rpm. Supernatant was discarded and it was, washed in phosphate-buffered saline (PBS) plus 10% Fetal Bovine Serum (FBS) for 10 min at 1200 rpm. Cells were collected and resuspended in 1 mL PBS with 10% FBS. In a cell population dot plot graph, MNC were gated for all flow cytometry analysis as shown in Figure 7.

### 3.5. Measurement of DNA Damage by the Comet Assay

DNA damage in MNC was analyzed by the alkaline comet assay as described by Singh *et al.* [39] with minor modifications. Regular microscope slides were precoated with 200 μL of 1.5% normal melting point agarose (in distilled water, 60 °C) (Sigma-Aldrich, St. Louis, MO, USA) and dried overnight at room temperature and then stored at 4 °C until use. Subsequently, 2 × 10^4^ MNC mixed with 100 μL of 0.5% low melting point agarose (in PBS, at 37 °C) (Invitrogen, Spain) were spread on the agarose-coated slides using a coverslip. After gelling at 4 °C for 20 min, coverslip was removed and the slides immersed in freshly prepared cold lysis solution (2.5 M NaCl, 100 mM EDTA, 10 mM Tris, adjusted at pH 10–10.5, with freshly added 1% Triton X-100 and 10% DMSO) at 4 °C for 1 h. After a 5 min washing in cold distilled water, slides were placed in the electrophoresis chamber, which was then filled with freshly-made alkaline buffer (300 mM NaOH, 1 mM EDTA, pH > 13) for 20 min at 4 °C. Electrophoresis was performed at 300 mA and 25 V for 30 min. All these steps were conducted without direct light in order to prevent additional DNA damage. Slides were then washed three times of 5 min each with 0.4 M Tris buffer, pH 7.5, to neutralize the excess alkali. Finally, 100 μL of ethidium bromide (20 μg/mL, Sigma-Aldrich) was added to each slide, covered with a coverslip and analyzed at a magnification of 20× using a fluorescence microscope (Olympus BX60, Essex, UK) equipped with excitation (510–550 nm) and barrier (590 nm) filters.

The DNA damage was evaluated using a visual classification of comets into five levels, according to the comet tail size, from 0 (undamaged with no tail) to 4 (maximally damaged with long tail). The extent of DNA damage was expressed in arbitrary units (a.u.). Images of 300 randomly selected cells (100 cells from each of three replicate slides) were analyzed from each animal and consequently generated three values of a.u. and the final result per animal was the average of these values. The damage index (DI) of the group ranged from 0 (all cells undamaged: 300 cells × 0) to 1200 (all cells with maximally damaged: 300 cells × 4). Score was evaluated by the summatory of multiplying the class of the comet by the number of cells with that damage. The damage frequency (%) was calculated using percentage of damage in each class.

### 3.6. Measurement of Cytoplasmatic Reactive Oxygen Species by DHE and DCFs

Dihydroethidium (DHE) and 2′,7′-dichlorofluorescein diacetate (DCF-DA) were used for the flow cytometric detection of intracellular superoxide anions (·O_2_^−^) and hydrogen peroxide (H_2_O_2_). DHE is freely permeable to cells and is rapidly oxidized, mostly by superoxide, to ethidium which binds to DNA and amplifies red fluorescence signal. DCF-DA is a cell-permeant indicator for H_2_O_2_ production that is nonfluorescent until oxidation occurs within the cell converting it to the fluorescent form, DCF, which remains trapped in the cell. To estimate the content of ·O_2_^−^ or H_2_O_2_ in cell suspension, 10^6^ MNC were incubated with 20 μL of DHE (160 μM) and 20 μL of DCF-DA (20 mM) for 30 min at 37 °C in the dark to load the cells with the dyes. For positive control, samples were treated for 5 min with 50 μM H_2_O_2_ to create an oxidative stress without being toxic to the cells, whereas for negative control the cells were incubated with ethanol. Cells were then washed, resuspended in PBS, and kept on ice for an immediate detection by flow cytometry (FACSCanto II, Becton Dickinson, San Juan, CA, USA). Data was acquired and analyzed using the FACSDiva software (Becton Dickinson, San Juan, CA, USA). For quantification of DHE and DCF fluorescence, samples were acquired in triplicate and 10,000 events were used for each measurement. Cells were excited at 488 nm and DHE and DCF fluorescence were detected using, respectively, 585/42 and 530/30 bandpass filters and data expressed as the median fluorescence intensity (MFI).

The bone marrow cells were gated based on 90° and forward-angle light scatter for MNC morphological identification according to Feng *et al.* [66]. Considering that these cells are small and less complex, size and granularity parameters were used to separate and analyze MNC population. MNC percentages were similar between apoE^−/−^ groups (Aged: 14.1 ± 0.73 *vs.* Young: 17.8 ± 2.18%) and C57 groups (Aged: 15.2 ± 0.91 *vs.* Young: 13.7 ± 1.3), indicating that aging and atherosclerosis did not change the fractions of MNC.

### 3.7. Apoptosis in MNC

For apoptosis assay aliquots of 200 μL were removed from the cells and centrifuged for 10 min at 1200 rpm and then resuspended in binding buffer 1 × (10 mmol HEPES, NaOH, pH 7.4, 140 mmol NaCl, 2.5 mmol CaCl_2_) at concentration of 1 × 10^6^ cells/mL. Then, 100 mL of this solution (1 × 10^5^ cells) were transferred to a new tube, where they received 2.5 μL of FITC-annexin V and 2.5 μL of propidium iodide (PI). The cells were incubated for 15 min at room temperature (25 °C) in the absence of light. Finally, 400 μL of 1× binding buffer was added to each tube and the samples analyzed by flow cytometry on a maximum period of 1 h. Next, the samples were analyzed using the FACSCANTO II flow cytometer (Becton Dickinson) to quantify the apoptotic rate in Q1 (annexin negative, but PI positive, which indicates cell damage), Q2 (annexin/PI double positive, which indicates late apoptotic cells), Q3 (annexin/PI double negative, which indicates live cells) and Q4 (annexin positive, but PI negative, which indicates early apoptotic cells). The apoptotic rate was determined as the percentage of Q2 + Q4.

### 3.8. Statistical Analysis

All data are expressed as the mean ± SEM. The normality (Gaussian distribution) of the variables was previously analyzed using the Kolmogorov-Smirnov test. When this test was significant, the statistical analysis was performed using the two-way analysis of variance (ANOVA). When the ANOVA showed significant differences, the Fisher’s test was performed as a *post hoc* analysis. When the test of normality was not significant, the statistical analysis was performed using the nonparametric Kruskal-Wallis test followed by the Dunn’s *post hoc* test to compare pairs of groups. The differences between means were considered significant when *p* < 0.05.

## 4. Conclusions

The present study focused on the bone marrow, a major source of production of cells. By using techniques of flow cytometry and comet assay, we were able to demonstrate a correlation between aging and augmented production of ROS, a condition that was aggravated by hypercholesterolemia. This study is the first to demonstrate the impact of hypercholesterolemia associated with aging process on production of ROS, DNA damage and apoptosis in bone marrow mononuclear cells in apoE^−/−^ animals.
